# Elevated gonadotropins and risk of dementia in Chinese adults aged over 80: a cross-sectional study

**DOI:** 10.3389/fnagi.2025.1651723

**Published:** 2025-10-17

**Authors:** Yunxia Zhu, Youyi Tu, Chenxi Ren, Yingying Ke, Qihao Guo

**Affiliations:** Department of Gerontology, Shanghai Sixth People’s Hospital Affiliated to Shanghai Jiao Tong University School of Medicine, Shanghai, China

**Keywords:** follicle-stimulating hormone, luteinizing hormone, Alzheimer’s disease-related dementia, apolipoprotein E epsilon 4, adults aged over 80, cross-sectional study

## Abstract

**Introduction:**

Age-related elevation of gonadotropins may contribute to cognitive decline, while apolipoprotein E epsilon 4 (APOE ε4) is an established risk factor for Alzheimer’s disease (AD). This study investigated the associations of follicle-stimulating hormone (FSH) and luteinizing hormone (LH) levels with global cognition and dementia in adults aged over 80.

**Methods:**

A total of 509 adults (440 males and 69 females) were included in this cross-sectional analysis, comprising 337 with normal cognition (NC), 97 with Alzheimer’s disease-related dementia (AD-D), and 75 with vascular dementia (VD). Cognitive status was assessed using Mini-Mental State Examination (MMSE). Plasma gonadotropins and sex hormones were measured by chemiluminescence. Multivariable linear and logistic regression models, adjusted for potential confounders, were employed.

**Results:**

Follicle-stimulating hormone concentrations were significantly elevated in males with dementia and females with VD compared to NC. LH concentrations were significantly elevated in VD across sexes compared with NC. Neither estradiol nor total testosterone differed across groups. Continuous LH, rather than FSH, was significantly associated with MMSE scores in the total cohort and males after adjusting covariates (both *p* < 0.05). When dichotomized by median (19.9 IU/L for males; 67.1 IU/L for females), FSH was significantly associated with MMSE after further adjusting for LH (both *p* < 0.05). A significant interaction between high FSH and APOE ε4 carrier status on cognitive impairment was observed (*p* < 0.05). After multivariate adjustment including LH, elevated FSH was independently associated with higher AD-D risk both when defined by median split (total sample: OR = 3.109; males: OR = 3.597) and as a continuous variable (total: OR = 1.033; males: OR = 1.048). In contrast, higher continuous LH was linked to lower AD-D risk in the total cohort and males, regardless of FSH adjustment. Neither FSH nor LH concentrations were associated with VD risk after adjusting for covariates. The area under the receiver operating characteristic curve for FSH in predicting AD-D in males was 0.600, with an optimal cutoff value of 28.4 IU/L.

**Conclusion:**

Elevated FSH and reduced LH may be associated with poorer cognition and an increased risk of AD-D in very old Chinese adults, particularly in males.

## 1 Introduction

In China, the number of individuals aged over 80 years is rapidly increasing due to extended life expectancy, with the World Health Organization projecting this population to exceed 90 million by 2050 (Gao and Liu, 2021). This group bears the highest incidence of dementia, particularly Alzheimer’s disease-related dementia (AD-D) and vascular dementia (VD), imposing a growing burden on families and society in the absence of effective prevention or treatment (Li et al., 2022). Despite this urgency, the oldest old remain underrepresented in clinical and epidemiological research.

Sex hormones have long been studied in relation to cognitive function in older adults, but the clinical evidence for supplementation remains limited and inconclusive. Consequently, attention has shifted toward gonadotropins, particularly follicle-stimulating hormone (FSH) and luteinizing hormone (LH), which regulate the secretion of sex hormones. Recent experimental work demonstrated that FSH promotes AD-like pathology by acting directly on hippocampal and cortical neurons and, in apolipoprotein E epsilon 4 (APOE ε4) female mice, by activating the C/EBPβ/δ-secretase pathway (Xiong et al., 2022, 2023). Notably, FSH levels gradually rise with age in men but surge during perimenopause and remain elevated thereafter (Vermeulen, 1993), suggesting that very old adults may be particularly affected. Neuroimaging studies further indicate links between high FSH, amyloid-β burden, and reduced gray matter in AD-vulnerable regions of midlife women at risk for AD (Nerattini et al., 2023). LH has also been implicated in AD pathogenesis (Butler et al., 2024; Burnham and Thornton, 2015). Elevated LH may promote β-amyloid production and deposition in the brain (Bowen et al., 2004; Casadesus et al., 2006). Clinical investigations, however, have yielded inconsistent findings regarding circulating LH levels and cognitive outcomes, with some studies reporting associations between higher LH and increased risk of cognitive impairment (Short et al., 2001), whereas others have observed null or even protective effects (Lee et al., 2017; Hogervorst et al., 2004).

Therefore, in this cross-sectional study of Chinese adults aged 80–98 years, we examined the associations of plasma FSH and LH with global cognitive status and the risk of dementia (AD-D and VD), and their potential interaction with the ApoE ε4 genotype.

## 2 Materials and methods

### 2.1 Study population

This single-center, prospective observational study collected baseline data from January 2019 to December 2019, with follow-up currently ongoing. A total of 618 participants aged 80–98 years (81% male) were initially enrolled at the Sixth People’s Hospital Affiliated to Shanghai Jiao Tong University, Shanghai, China (clinical trial registration number: ChiCTR1800018015). Of these, 611 participants completed the Mini-Mental State Examination (MMSE) for cognitive assessment and underwent measurement of plasma reproductive hormone levels. Finally, a total of 509 individuals (440 males and 69 females) were included in this study. Participants with disorders of the hypothalamo-pituitary-gonadal axis or those receiving sex hormone replacement therapy, both of which could influence reproductive hormone levels, were excluded. The detailed inclusion and exclusion criteria have been described previously (Zhu et al., 2021; Ke et al., 2021). In addition, individuals with mild cognitive impairment were excluded to better address the specific aims of the present study. Baseline assessments included a physical examination, blood sample collection, and in-person interviews that gathered information on sociodemographic characteristics, lifestyle factors, cognitive function, and medical history. The study was approved by the Ethics Committee of Shanghai Jiao Tong University Affiliated Sixth People’s Hospital (Reference number: 2018–109) and was conducted in accordance with the principles of the Declaration of Helsinki. Written informed consent was obtained from all participants or their legal guardians.

### 2.2 Laboratory measurements

Following an overnight fast, peripheral venous blood samples were collected from each participant. Serum and plasma were separated and stored for analysis. The measurement of serum folate (reference range: 4.20–19.80 μg/L) and vitamin B12 (reference range: 197–771 ng/L) levels were conducted using the Roche Elecsys Immunoassay on the Cobas e601 analyzer. The levels of free triiodothyronine [FT3; detection range (DR): 0.4–50.0 pM; reference range: 3.1–6.8 pM], free thyroxine (FT4; DR: 0.3–100.0 pM; reference range: 12.0–22.0 pM), and thyroid-stimulating hormone (TSH; DR: 0.06–99.0 mIU/L; reference range: 0.27–4.20 mIU/L) were determined using electrochemiluminescence immunoassays (Roche Diagnostics GmbH). Sex hormones, including total testosterone (TT; DR: 0.087–52.000 nM; reference range: 6.68–25.70 nM) and E_2_ (DR: 18.35–15,781.00 pM; reference range: 41.4–159.0 pM), as well as pituitary gonadotropins—FSH (DR: 0.5–160.0 IU/L; reference range: 1.5–12.4 IU/L) and LH (DR: 0.1–200.0 IU/L; reference range: 1.7–8.6 IU/L) were measured using chemiluminescence assays (Abbott GmbH & Co. KG). All assays demonstrated inter-assay and intra-assay coefficients of variation below 10%. Four samples with values below the detection limits for LH, E_2_, or TT were re-analyzed using the respective lower detection limits. APOE genotyping was conducted using polymerase chain reaction followed by direct sequencing. Participants were categorized based on APOE ε4 status (carriers vs. non-carriers).

### 2.3 Assessment of global cognition function and dementia diagnosis

Global cognitive function was assessed using the MMSE, a widely used tool consisting of multiple simple tasks, with a maximum score of 30 points. All-cause dementia (ACD) was diagnosed complying with the 2011 criteria established by the National Institute on Aging–Alzheimer’s Association (NIA-AA) workgroups (Mckhann et al., 2011). AD-D was further identified based on NIA-AA criteria and confirmed through cranial magnetic resonance imaging (cMRI). VD was diagnosed if cognitive impairment occurred in association with stroke or cerebrovascular lesions, provided dementia developed within 3–6 months post-event and persisted for at least 3 months.

### 2.4 Covariates

Data on demographic characteristics (age, sex, and years of education) and comorbidities known to be strongly associated with cognitive function, such as diabetes, hypertension, and depression, were collected through self-report and verified by medical records. Lifestyle risk factors, including smoking and alcohol consumption, were also recorded. Due to the relatively low number of current or former smokers and drinkers, only the prevalence of non-smoking and non-drinking was reported. Waist circumference (WC) was measured at the midpoint between the lower rib margin and the iliac crest along the midaxillary line, with participants standing in a relaxed posture while wearing underwear.

### 2.5 Statistical analysis

The Shapiro-Wilk test was used to assess the normality of variable distributions. Normally distributed variables were presented as mean ± standard deviation and compared between groups using the Student’s *t*-test, whereas skewed variables were reported as median (25th–75th percentile) and compared between groups using Mann-Whitney *U*-test. Additionally, categorical variables were presented with counts and percentages, and compared using chi-square test. Linear regression models were used to examine the association between plasma gonadotropins, either as a continuous variable or as a dichotomous variable (based on a median split), and MMSE scores. Binary logistic regression models were adopted to explore the link between gonadotropins and the risk of dementia, including ACD, AD-D, and VD. Crude and adjusted odds ratios (ORs) with 95% confidence intervals (CIs) were calculated. The linear and logistic regression models were adjusted for covariates that may influence gonadotropins and cognitive function (age, years of education, history of hypertension or depression, WC, TT, E_2_, and FT3), and additionally for LH or FSH, respectively, to clarify whether the associations remained independent of the other gonadotropin. Two-way analysis of variance (ANOVA) was performed to assess the interaction between gonadotropins and APOE ε4 status on cognitive performance, adjusting for age and education. Receiver operating characteristic (ROC) curve analysis was performed to determine optimal cut-off value of FSH for diagnosing AD-D in males, and the area under the ROC curve (AUROC) with 95% CI was calculated. The optimal cutoff point was identified using the maximum Youden Index. All statistical analyses were performed using SPSS for Windows, version 23.0, with figures generated using GraphPad Prism version 8.0. Statistical significance was defined as a two-tailed *p*-value less than 0.05.

## 3 Results

### 3.1 Baseline characteristics

A total of 172 elderly participants (33.8%) were classified as having ACD, of whom 97 (56.4%) were diagnosed with AD-D and 75 (43.6%) with VD. MMSE scores were significantly lower in both the AD-D and VD groups compared to the normal cognition (NC) group (both *p* < 0.01). Table 1 shows baseline characteristics of the different groups. Compared to participants with NC, individuals with AD-D or VD were older, had fewer years of education, higher prevalence of depression, and lower FT3 levels (all *p* < 0.01). In addition, the prevalence of hypertension was significantly lower in the AD-D group in contrast with the NC group (*p* < 0.01). No significant differences were observed among the groups regarding sex, proportion of non-smokers and non-drinkers, APOE ε4 carrier status, prevalence of diabetes, WC, or levels of folate, vitamin B12, FT4, and TSH.

**TABLE 1:** Characteristics of the studied population by cognitive status.

Variable	Normal cognition (*n* = 337)	Alzheimer’s dementia (*n* = 97)	Vascular dementia (*n* = 75)
Age (y)	85 (83–88)	90 (86–93)**	89 (84–92)**
Male (%, *n*)	87.8 (296)	78.4 (76)	90.7 (68)
WC (cm)	91 (85–97)	89 (83-95)	90 (83–95)
Education (year)	16 (16–16)	16 (9–16)**	16 (9–16)**
Non-smoker (%, *n*)	78.0 (263)	77.3 (75)	65.3 (49)
Non-drinker (%, *n*)	85.2 (287)	84.5 (82)	78.7 (59)
Hypertension (%, *n*)	86.9 (293)	74.2 (72)**	88.0 (66)
Diabetes (%, *n*)	37.1 (125)	36.1 (35)	45.3 (34)
Depression (%, *n*)	1.8 (6)	7.4 (7)**	10.0 (7)**
APOE ε4 (%, *n*)^#^	11.7 (27)	11.6 (8)	22.6 (14)
Folate (μg/L)	8.0 (5.7–14.1)	7.7 (5.5–14.0)	8.1 (5.1–17.4)
Vitamin B12 (ng/L)	605 (420–945)	600 (399–939)	690 (487–1093)
FT3 (pM)	4.0 ± 0.6	3.7 ± 0.7**	3.8 ± 0.6**
FT4 (pM)	16.3 ± 2.3	16.4 ± 2.5	16.4 ± 2.7
TSH (mIU/L)	2.4 (1.6–3.7)	2.6 (1.7–4.3)	2.6 (1.7–4.1)
MMSE score	28 ± 2	13 ± 4**	15 ± 4**

^#^A total of 361 subjects having the data of APOE ε4 genotype.

*p < 0.05, **p < 0.01 against non-dementia. APOE, apolipoprotein E; FT3, free triiodothyronine; FT4, free thyroxine; MMSE, mini-mental status examination; TSH, thyroid-stimulating hormone; WC, waist circumference.

### 3.2 Plasma gonadotropins levels were elevated in subjects with dementia

**FIGURE 1:**
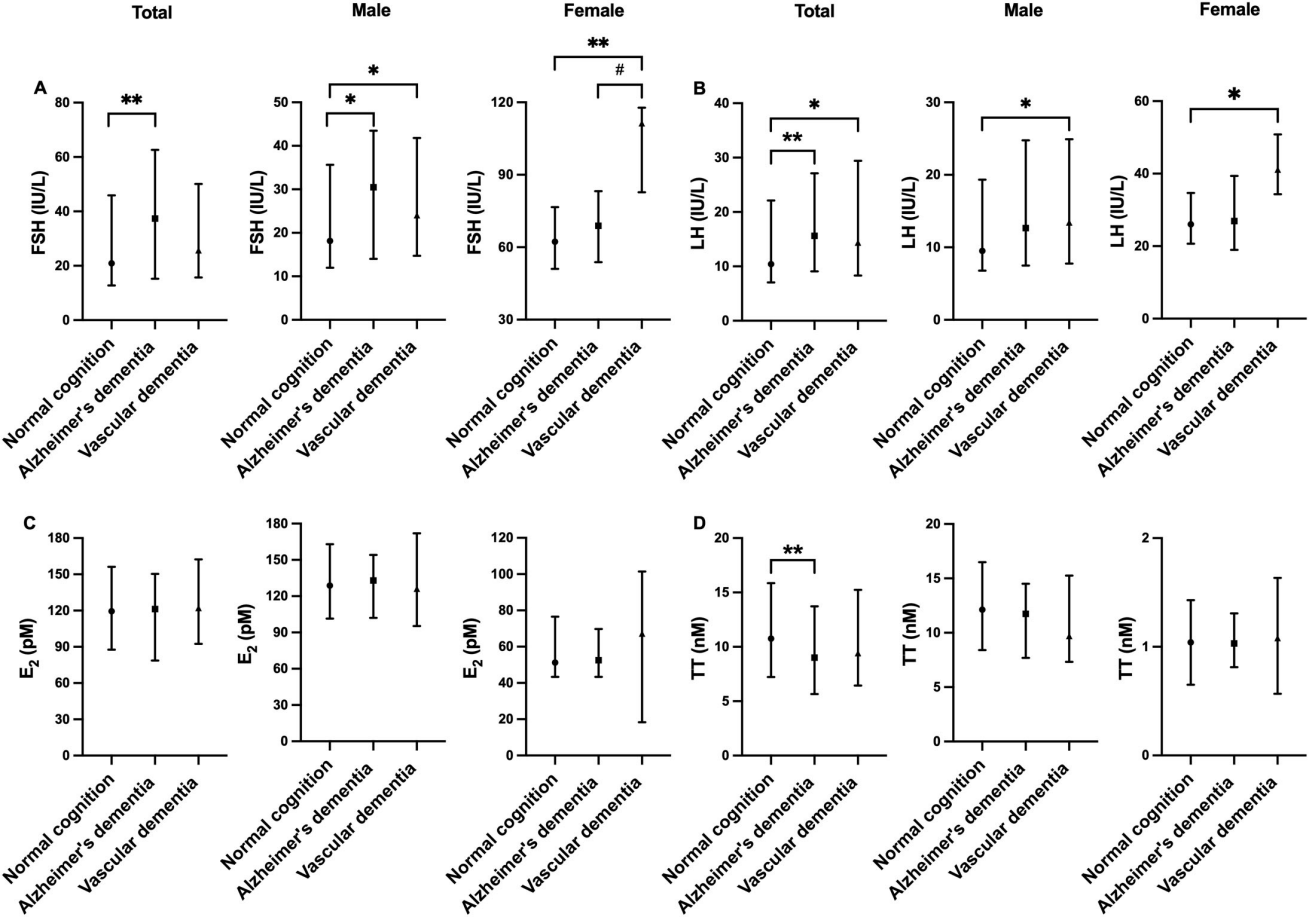
Comparison of FSH **(A)**, LH **(B)**, E2 **(C)**, and TT **(D)** levels among normal cognition, Alzheimer’s disease-related dementia, and vascular dementia groups. Error bars show median (25th percentile to 75th percentile). *p < 0.05, **p < 0.01 against normal cognition, ^#^*p* < 0.05 Alzheimer’s dementia vs. Vascular dementia. E_2_, estradiol; FSH, follicle-stimulating hormone; LH, luteinizing hormone; TT, total testosterone.

The levels of reproductive hormones across different groups are summarized in Supplementary Table 1 and illustrated in Figure 1. FSH and LH levels were significantly higher in the VD group compared to the NC group in both sexes (all *p* < 0.05). Among males, FSH levels were also significantly higher in the AD-D group in contrast with the NC group (*p* < 0.05). In females, FSH levels were higher in the VD group when compared to the AD-D group (*p* < 0.05). TT and E_2_ levels were not different with statistical significance among the groups.

### 3.3 Association between gonadotropins and MMSE score

Given the observed differences in FSH and LH levels among groups with varying cognitive status, the association between gonadotropins and MMSE scores was further analyzed. After adjusting for age, years of education, history of hypertension and depression, WC, FT3, E_2_, TT and APOE ε4 genotype, no significant association was found between continuous FSH levels and MMSE scores in the linear regression model. Given the skewed distribution of FSH levels, we further dichotomized it into “high” and “low” groups based on sex-specific median values (male: 19.9 IU/L; female: 67.1 IU/L); however, no significant association was observed in the multiple linear regression model. Notably, a significant association emerged after additional adjustment for LH (the total population: β = −2.126, *p* = 0.030; males: β = −2.719, *p* = 0.015). In contrast, continuous LH levels were positively associated with MMSE scores in the total population and in males regardless of FSH adjustment, whereas dichotomized LH (male: 10.1 IU/L; female: 27.4 IU/L) showed no significant association (Table 2). Collectively, these findings suggest a threshold effect of FSH and a linear inverse association of LH with cognitive function.

**TABLE 2:** Multiple linear regression analyses of association between gonadotropins and mini-mental status examination (MMSE) scores in the total, male and female populations.

Models	Variables	Groups	β	95% CI	SE	*P*-value
Model 1	Continuous FSH	Total	0.014	−0.022 to 0.050	0.018	0.446
		Male	0.024	−0.015 to 0.063	0.020	0.230
		Female	0.014	−0.119 to 0.146	0.065	0.836
	Dichotomized FSH	Total	−0.701	−2.380 to 0.978	0.853	0.412
		Male	−0.561	−2.397 to 1.275	0.933	0.548
		Female	−1.481	−6.260 to 3.299	2.352	0.533
	Continuous LH	Total	0.084	0.010 to 0.158	0.038	**0.026**
		Male	0.108	0.029 to 0.186	0.040	**0.007**
		Female	0.084	−0.138 to 0.306	0.109	0.446
	Dichotomized LH	Total	−0.274	−1.986 to 1.438	0.870	0.753
		Male	−0.076	−1.929 to 1.777	0.941	0.936
		Female	1.110	−3.827 to 6.048	2.429	0.651
Model 2	Continuous FSH	Total	−0.043	−0.101 to 0.015	0.029	0.144
		Male	−0.048	−0.112 to 0.015	0.032	0.137
		Female	−0.033	−0.210 to 0.143	0.087	0.702
	Dichotomized FSH	Total	−2.126	−4.045 to -0.207	0.976	**0.030**
		Male	−2.719	−4.905 to 0.533	1.111	**0.015**
		Female	−3.290	−8.821 to 2.242	2.719	0.235
	Continuous LH	Total	0.149	0.034 to 0.264	0.058	**0.011**
		Male	0.181	0.057 to 0.306	0.063	**0.004**
		Female	0.121	−0.176 to 0.419	0.146	0.414
	Dichotomized LH	Total	−0.779	−2.709 to 1.151	−0.046	0.428
		Male	−1.026	−3.232 to 1.180	1.121	0.361
		Female	1.296	−5.110 to 7.703	3.149	0.683

Statistically significant comparisons are bolded. Model 1: adjusted for age, years of education, history of hypertension and depression, waist circumference, free triiodothyronine (FT3), estradiol (E_2_), total testosterone (TT) and apolipoprotein E (APOE) genotype. Model 2: Model 1 + additionally adjusted for FSH or LH. FSH dichotomy: male, 19.9 IU/L; female, 67.1 IU/L. LH dichotomy: male, 10.1 IU/L; female, 27.4 IU/L. FSH, follicle-stimulating hormone; LH, luteinizing hormone.

### 3.4 Interaction of gonadotropins and APOE ε4 in cognition impairment

**FIGURE 2:**
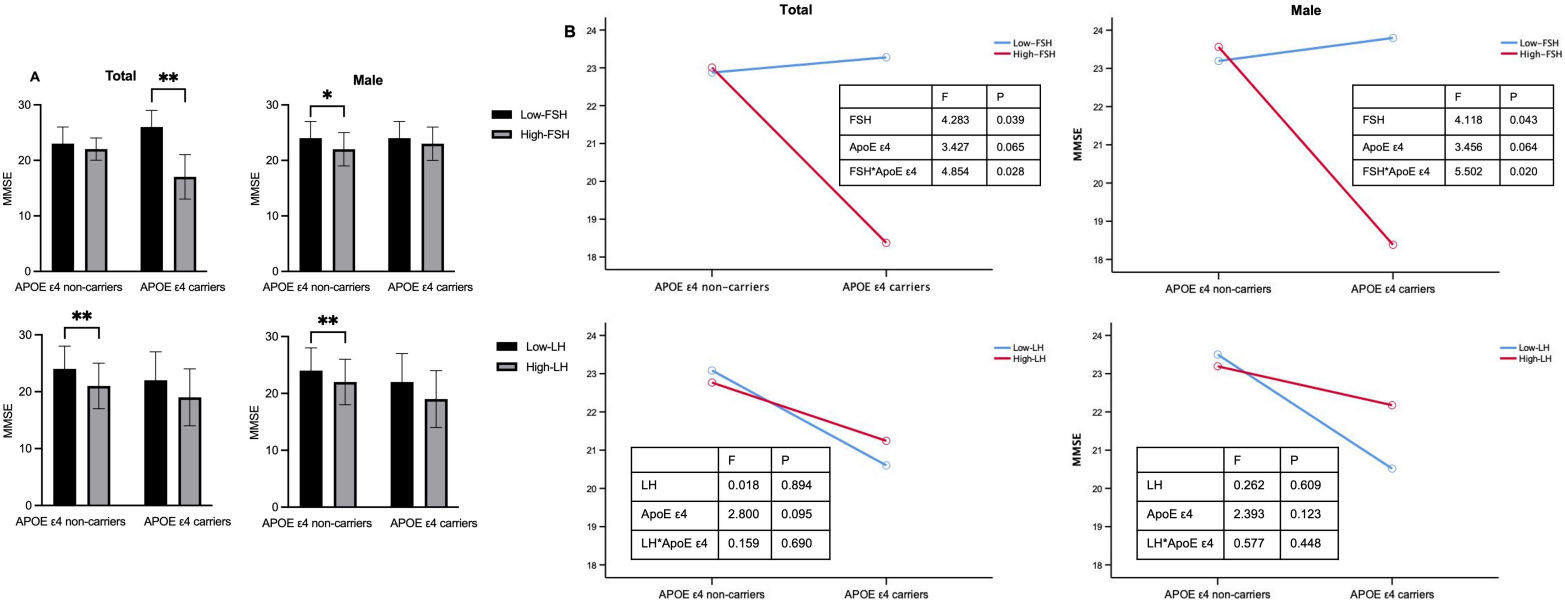
The impact of APOE ε4 and gonadotropins on cognitive function. **(A)** MMSE scores in APOE ε4 non-carriers and carriers for the total cohort and males. **(B)** Interactive effects of gonadotropin levels and APOE ε4 status on MMSE scores in the total and male cohorts. Age and education year were controlled in the two-way analysis of variance (ANOVA). *p < 0.05, **p < 0.01 compared with Low-FSH or Low-LH group. Low-FSH < 19.9 IU/L (male) or < 67.1 IU/L (female). APOE, apolipoprotein E; FSH, follicle-stimulating hormone; MMSE, mini-mental status examination.

Considering the additive effect of FSH and APOE ε4 in the development of AD-like pathogenesis in female mice (Xiong et al., 2023), we investigated whether gonadotropins and APOE ε4 interact in cognition impairment. After adjusting for age and education, the MMSE scores were significantly influenced by the interaction between FSH and APOE ε4 in both the total cohort and male participants (Figure 2 and Table 3). Among APOE ε4 carriers, participants in the high-FSH subgroup had significantly lower MMSE scores than those in the low-FSH subgroup (*p* < 0.01). In male APOE ε4 carriers, a similar decreasing trend was observed (*p* = 0.053) (Table 3). We also analyzed the interaction between LH and APOE genotype on MMSE, and found no significant effect. Interestingly, in APOE ε4 non-carriers, the high-LH group exhibited lower MMSE scores than the low-LH group, both in the total population and in males (*p* < 0.01). Due to the small sample size and low number of APOE ε4 carriers among females, the results are unreliable and the power is low. Therefore, the analysis was not conducted in females.

**TABLE 3:** Interactive effects of gonadotropins and APOE ε4 on cognitive function in older adults.

Groups	Variable	APOE ε 4 non-carriers		APOE ε 4 carriers		Two-way ANOVA					
		Low-FSH	High-FSH	Low-FSH	High-FSH	APOE		FSH		APOE × FSH	
						*F*	*P*	*F*	*P*	*F*	*P*
Total	MMSE	23 ± 3	22 ± 2	26 ± 3	17 ± 4**	3.427	0.065	4.283	0.039	4.854	**0.028**
Male	MMSE	24 ± 3	22 ± 3*	24 ± 3	23 ± 3	3.456	0.064	4.118	0.043	5.502	**0.020**
**Groups**	**Variable**	**Low-LH**	**High-LH**	**Low-LH**	**High-LH**	**APOE**		**LH**		**APOE × FSH**	
						***F***	***P***	***F***	***P***	***F***	***P***
Total	MMSE	24 ± 4	21 ± 4**	22 ± 5	19 ± 5	2.800	0.095	0.018	0.894	0.159	0.690
Male	MMSE	24 ± 4	22 ± 4**	22 ± 5	19 ± 5	2.393	0.123	0.262	0.609	0.577	0.448

Statistically significant comparisons are bolded. A total of 361 subjects having the data of APOE ε4 genotype. The data were presented as mean ± standard deviation. Age and education year were controlled in the two-way ANOVA. FSH dichotomy: male, 19.9 IU/L; female, 67.1 IU/L. LH dichotomy: male, 10.1 IU/L; female, 27.4 IU/L.

*p < 0.05, **p < 0.01 compared with Low-FSH or Low-LH group. APOE, apolipoprotein E; FSH, follicle-stimulating hormone; LH, luteinizing hormone.

### 3.5 Association of higher gonadotropins levels with the risk of AD-D

**FIGURE 3:**
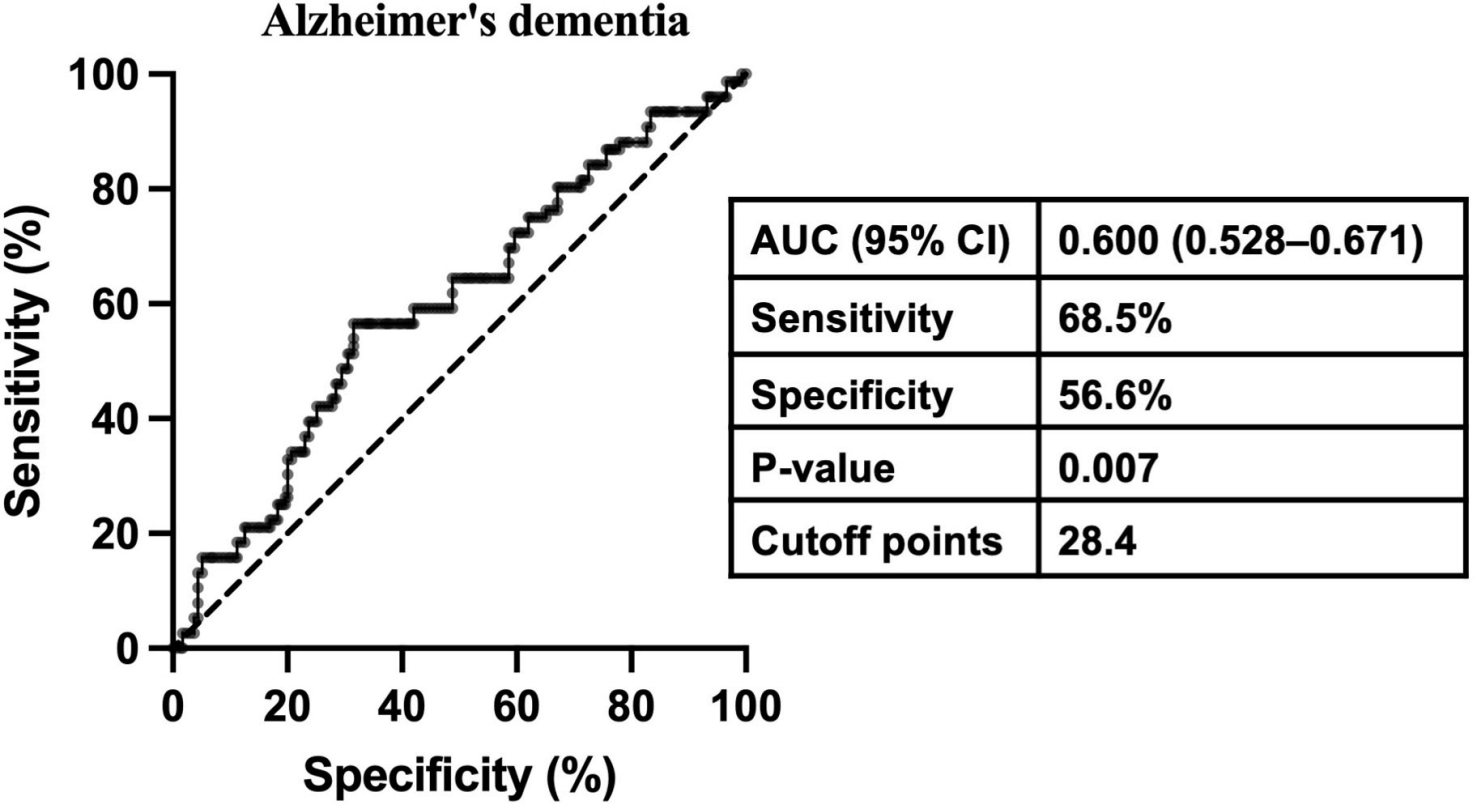
Receiver operating characteristic curve analysis determining the optimal cutoff point of FSH for Alzheimer’s disease-related dementia in older males. AUC, area under the receiver operating characteristic curve; CI: confidence interval.

The relationship between gonadotropins levels and dementia is presented in Table 4. The proportion of subjects with ACD, AD-D, or VD was higher in the high-FSH group in contrast with the low-FSH group. Binary logistic regression analysis revealed that neither continuous FSH nor dichotomized FSH was associated with the risk of AD-D after adjusting for age, years of education, history of hypertension and depression, WC, FT3, E_2_, TT, and APOE ε4 genotype. However, after further adjusting for LH, both continuous FSH and dichotomized FSH were significantly associated with an increased risk of AD-D in the total population (continuous FSH, OR: 1.033, 95% CI: 1.004–1.063; dichotomized FSH, OR: 3.109; 95% CI: 1.276–7.576) and males (continuous FSH, OR: 1.048, 95% CI: 1.011–1.086; dichotomized FSH, OR: 3.597; 95% CI: 1.159–11.166). In the overall cohort, dichotomized FSH was also associated with an increased risk of ACD (OR: 2.092, 95% CI: 1.099–3.983). To assess the role of FSH in predicting the risk of AD-D in older males, ROC curve analysis was performed. As shown in Figure 3, FSH demonstrated an AUROC of 0.600 (95% CI: 0.528–0.671; *p* < 0.01), with an optimal cutoff value of 28.4 IU/L, achieving a sensitivity of 68.5% and a specificity of 56.6% for AD-D. For LH, continuous but not dichotomized LH was significantly associated with a decreased risk of AD-D in the total and males populations regardless of adjusting for FSH. No significant association was observed between gonadotropins and VD. Due to the limited number of female participants and dementia cases across subgroups, the regression models were unstable and the results unreliable; therefore, corresponding analyses were not conducted in females.

**TABLE 4:** Binary logistic regression analysis of associations between gonadotropins and dementia in older adults.

All (*n* = 509)	Prevalence (%, *n*)	Models (OR, 95% CI)		
		Crude model	Adjusted model 1	Adjusted model 2
**All-cause dementia**				
High-FSH	41.1 (106)**	1.955 (1.344–2.844)**	1.455 (0.840–2.522)	2.092 (1.099–3.983)*
Low-FSH	26.3 (66)	1.00	1.00	1.00
FSH	–	1.012 (1.005–1.020)**	0.998 (0.986–1.010)	1.011 (0.993–1.030)
High-LH	41.9 (106)**	2.076 (1.426–3.021)**	1.027 (0.588–1.795)	1.142 (0.613–2.127)
Low-LH	25.8 (66)	1.00	1.00	1.00
LH	–	1.029 (1.014–1.044)**	0.985 (0.961–1.009)	0.971 (0.934–1.009)
**Alzheimer’s dementia**				
High-FSH	26.9 (56)*	1.662 (1.053–2.624)*	1.353 (0.661–2.771)	3.109 (1.276–7.576)*
Low-FSH	18.1 (41)	1.00	1.00	1.00
FSH	–	1.014 (1.005–1.023)**	0.995 (0.979–1.012)	1.033 (1.004–1.063)*
High-LH	29.3 (61)**	2.190 (1.376–3.486)**	1.031 (0.497–2.139)	1.234 (0.541–2.814)
Low-LH	15.9 (36)	1.00	1.00	1.00
LH	–	1.027 (1.008–1.045)**	0.961 (0.929–0.995)*	0.916 (0.864–0.972)**
**Vascular dementia**				
High-FSH	24.8 (50)**	2.434 (1.439–4.118)**	1.682 (0.836–3.386)	1.793 (0.794–4.050)
Low-FSH	11.9 (25)	1.00	1.00	1.00
FSH	–	1.010 (1.001–1.019)*	1.003 (0.989–1.017)	1.004 (0.983–1.025)
High-LH	23.4 (45)*	1.939 (1.165–3.228)*	1.115 (0.545–2.285)	1.136 (0.508–2.539)
Low-LH	13.6 (30)	1.00	1.00	1.00
LH	–	1.031 (1.012–1.051)**	1.008 (0.978–1.038)	1.097 (0.963–1.054)
**Male (*n* = 440)**				
**All-cause dementia**				
High-FSH	39.7 (89)**	1.930 (1.285–2.898)**	1.206 (0.647–2.247)	1.759 (0.830–3.726)
Low-FSH	25.5 (55)	1.00	1.00	1.00
FSH	–	1.012 (1.003–1.021)**	0.997 (0.984–1.010)	1.103 (0.992–1.035)
High-LH	41.1 (90)**	2.158 (1.435–3.245)**	0.840 (0.444–1.589)	0.955 (0.456–1.998)
Low-LH	24.4 (54)	1.00	1.00	1.00
LH	–	1.028 (1.011–1.046)**	0.982 (0.956–1.008)	0.965 (0.924–1.006)
**Alzheimer’s dementia**				
High-FSH	25.0 (45)*	1.731 (1.038–2.887)*	1.169 (0.482–2.837)	3.597 (1.159–11.166)*
Low-FSH	16.1 (31)	1.00	1.00	1.00
FSH	–	1.012 (1.001–1.023)*	0.994 (0.976–1.012)	1.048 (1.011–1.086)*
High-LH	28.3 (51)**	2.641 (1.553–4.490)**	0.911 (0.375–2.216)	1.244 (0.441–3.506)
Low-LH	13.0 (25)	1.00	1.00	1.00
LH	—	1.023 (1.001–1.046)*	0.954 (0.916–0.992)*	0.882 (0.817–0.953)**
**Vascular dementia**				
High-FSH	24.6 (44)**	2.186 (1.265–3.780)**	1.396 (0.659–2.956)	1.379 (0.553–3.437)
Low-FSH	13.0 (24)	1.00	1.00	1.00
FSH	–	1.011 (1.000–1.022)	1.003 (0.988–1.018)	1.005 (0.982–1.029)
High-LH	23.2 (39)*	1.741 (1.022–2.966)*	0.876 (0.401–1.914)	0.831 (0.331–2.087)
Low-LH	14.8 (29)	1.00	1.00	1.00
LH	–	1.032 (1.011–1.054)**	1.006 (0.976–1.037)	1.004 (0.957–1.053)

Data were a proportion with a number for categorical variables and odds ratios (OR) with 95% confidence interval (CI). Model 1: adjusted for age, years of education, history of hypertension and depression, waist circumference, free triiodothyronine (FT3), estradiol (E_2_), total testosterone (TT) and apolipoprotein E (APOE) genotype. Model 2: Model 1 + additionally adjusted for FSH or LH. FSH dichotomy: male, 19.9 IU/L; female, 67.1 IU/L. LH dichotomy: male, 10.1 IU/L; female, 27.4 IU/L. FSH, follicle-stimulating hormone; LH, luteinizing hormone.

*p < 0.05, **p < 0.01 compared with Low-FSH or Low-LH group.

## 4 Discussion

This study found that higher FSH levels were associated with poorer global cognitive performance and an increased risk of dementia, particularly AD-D, independent of LH adjustment. Moreover, the interaction between elevated FSH levels and APOE ε4 carrier status contributed to a greater risk of cognitive decline. In contrast, LH appeared to be inversely associated with the risk of AD-D, particularly in males, suggesting a protective effect. These findings provide new insights into the role of gonadotropins in cognitive health, particularly in oldest-old adults.

Dementia is a leading cause of disability in older adults, particularly those aged over 80. For the first time, this study investigated the link between FSH and dementia in this often-overlooked age group, and we found that elevated FSH levels were correlated with a higher risk of AD-D. We observed increased FSH levels in male AD-D patients compared to NC participants, which partially aligns with a previous study reporting higher FSH levels in Chinese males over 65 years old with AD compared to age-matched subjects with amnestic mild cognitive impairment (Li et al., 2020). Another study also reported a negative association between FSH and executive function (Castanho et al., 2014). However, two large cross-sectional studies, with 3,369 and 981 men, respectively, found no significant association, possibly due to their wider age ranges (40–79 and 48–80 years) (Lee et al., 2010; Fonda et al., 2005). Indeed, one study suggested that elevated FSH levels were observed only in elderly AD patients (average age 80 years), but not in younger cases (average age 66 years), when compared with controls (Hogervorst et al., 2004). The exact effect of FSH on the progression of AD in humans remains unclear. However, animal studies have shown that FSH directly affects hippocampal and cortical neurons, exacerbating AD-related pathology, and that neutralizing FSH can reverse AD-like symptoms in mouse models (Xiong et al., 2022). Moreover, inhibiting FSH signaling by depleting the FSH receptor protects against the progression of spatial memory deficits in mice (Korkmaz et al., 2025). These findings support a potential role for FSH in the development and progression of AD. APOE ε4 is widely acknowledged as a genetic risk factor for AD. Our study also highlighted an interaction between FSH and APOE ε4 in cognitive impairment: in APOE ε4 carriers, the adverse effect of high FSH on cognition was more pronounced, partially consistent with the results from animal studies (Xiong et al., 2023). Importantly, unlike prior studies restricted to females, we observed this interaction in both the overall cohort and in males, extending evidence of FSH–APOE ε4 interaction beyond female models. Unlike prior animal studies, we didn’t observe this interaction in female participants, possibly due to the small sample size (data not shown). Further research is needed to investigate the FSH-APOE ε4 interaction in females, particularly given their synergistic activation of the C/EBPβ/δ-secretase pathway in female mice (Xiong et al., 2023) and the association between elevated FSH and AD biomarkers in midlife women (Nerattini et al., 2023). A key finding in our study was that the association between FSH and cognitive impairment became significant only after adjusting for LH. This suggests that LH may mediate the relationship between FSH and cognition. Both FSH and LH influence cognitive function through distinct pathways, with LH affecting testosterone levels, which are known to have neuroprotective effects (van Beurden et al., 1976; Ascoli et al., 2002). The absence of a significant association between FSH and cognition before adjusting for LH may reflect the complex interplay between these two hormones. This emphasizes the need to consider both gonadotropins when examine their combined effect on cognitive health in aging populations.

Our study also revealed that LH was inversely associated with the risk of AD-D, particularly in males, while its role in females was not investigated due to sample size limitations. This finding contrasts with some previous studies reporting a neutral or detrimental role of LH on cognition (Lee et al., 2017; Fonda et al., 2005; Butler et al., 2024; Atwood and Bowen, 2015). This discrepancy may be attributed to several factors. First, our study focused on an older population (80–98 years), which may have allowed us to detect more distinct effects of LH on cognition compared to studies with broader age ranges (Lee et al., 2010; Fonda et al., 2005). Second, the potential neuroprotective effect of LH in males may be linked to its regulation of testosterone, known for its anti-inflammatory and neurotrophic properties (Yang et al., 2020; Janowsky, 2006). Third, our models controlled for a wide range of potential confounders, including APOE ε4 genotype, which was not consistently accounted for in earlier studies, thereby increasing the robustness of our findings. Moreover, recent genetic studies have shown that interactions between LH receptor (LHCGR) polymorphisms and APOE genotype may significantly influence AD risk in males. Specifically, the intronic LHCGR variant rs4073366 (lhcgr2) was found to markedly reduce AD risk among APOE ε4 carriers, effectively reversing the increased risk typically conferred by APOE ε4 (Haasl et al., 2008). These factors, along with the emphasis on sex-specific effects, could explain the differences observed between our results and those from prior research. Indeed, the contrasting effects of FSH and LH on cognitive functioning was reported before (Rodrigues et al., 2008). Nonetheless, further longitudinal studies are needed to confirm these findings and explore the mechanisms underlying LH’s protective role in cognitive health.

Although gonadotropins have been associated with vascular alterations and metabolic dysfunctions such as the blood-brain barrier permeability, obesity, insulin resistance, and dyslipidemia (Wilson et al., 2008; Liu et al., 2015; Song et al., 2016; Wang et al., 2017), our study found no direct associations with VD. A possible explanation is that gonadotropins may exert their effects through metabolic or neurodegenerative pathways, as suggested in AD, but these mechanisms may not play a central role in the ischemic and vascular injury processes underlying VD. Thus, gonadotropin-related alterations may be disease-specific, influencing AD risk but not directly contributing to VD.

This study has several strengths. Firstly, fasting blood samples were collected to minimize variations in reproductive hormone levels caused by circadian rhythms. Secondly, we adjusted for multiple potential confounders, including socio-demographic factors, lifestyle habits, comorbidities, biochemical indicators, and APOE genotype. Finally, we focused on participants within a narrow age range (80–98 years) to reduce the confounding effects of aging on cognition. However, there are some limitations. The cross-sectional design prevents any conclusions about causality between gonadotropins levels and cognitive decline. Despite adjusting for many known confounders, the influence of unknown or unmeasured factors—such as socioeconomic status, family history of AD, or prior hormone therapy use—cannot be ruled out. While our study population did not include individuals currently receiving hormone therapy, past exposure may still have affected gonadotropin levels and cognitive outcomes, and the absence of such detailed information may have introduced residual confounding. Additionally, cognitive assessment was restricted to global function; further investigation into the relationship between gonadotropins and specific cognitive domains is warranted. Finally, MMSE is less sensitive to subtle cognitive changes than other comprehensive tools. However, given the extreme aging populations in the current study, MMSE offered a practical and validated approach, though future work with more sensitive measures such as the Montreal Cognitive Assessment (MoCA) is warranted.

In conclusion, this study provides new evidence of a relationship between higher FSH levels and cognitive decline in individuals aged over 80. Our findings suggest that elevated FSH is associated with a greater risk of AD-D in males. However, due to large inter-individual variation and limited sensitivity and specificity in ROC analyses, FSH alone cannot be used as a predictive biomarker for AD-D. The synergistic interaction between elevated FSH levels and APOE ε4 carrier status exacerbate cognitive decline. Moreover, higher LH levels seem to have a protective role against AD-D in older males. Future studies should explore the interactions between FSH, LH, and other hormonal and genetic factors in both males and females to better understand their role in cognitive aging.

## Data Availability

The raw data supporting the conclusions of this article will be made available by the authors, without undue reservation.
