# Correction: Elevated gonadotropins and risk of dementia in Chinese adults aged over 80: a cross-sectional study

**DOI:** 10.3389/fnagi.2026.1859014

**Published:** 2026-05-05

**Authors:** Yunxia Zhu, Youyi Tu, Chenxi Ren, Yingying Ke, Qihao Guo

**Affiliations:** Department of Gerontology, Shanghai Sixth People's Hospital Affiliated to Shanghai Jiao Tong University School of Medicine, Shanghai, China

**Keywords:** follicle-stimulating hormone, luteinizing hormone, Alzheimer's disease-related dementia, apolipoprotein E epsilon 4, adults aged over 80, cross-sectional study

In the published article, in the **Funding** section, an incorrect grant number was provided for the funding source “Shanghai Traditional Chinese Medicine Scientific Research Project” as “[2024BJ001]”. The correct grant number for this funding source should have instead been “[2024BJ002]”. The corrected Funding section appears below.

## Funding

The author(s) declare that financial support was received for the research and/or publication of this article. This work was supported by Clinical Research Fund of Shanghai Sixth People's Hospital (ynlc201828), Shanghai Traditional Chinese Medicine Scientific Research Project (2024BJ002), and Basic Research Fund of Shanghai Sixth People's Hospital (ynms202208).

The original version of this article has been updated.

