# Supplementary material for: Elevated gonadotropins and risk of dementia in Chinese adults aged over 80: a cross-sectional study
Source: Front Aging Neurosci. 2025 Oct 17;17:1651723. doi: 10.3389/fnagi.2025.1651723 (PMC12575386; doi:10.3389/fnagi.2025.1651723)
Supplement: Supplementary file 1 [file Table_1.docx]

**TABLE S1 Levels of reproductive hormones in the population.**

| Groups | Variables | Normal cognition | Alzheimer's dementia | Vascular dementia |
| --- | --- | --- | --- | --- |
| Total (N) |  | 337 | 97 | 75 |
|  | FSH (IU/L) | 20 (13-45) | 37 (15-63) ^**^ | 25 (16-50) |
|  | LH (IU/L) | 10 (7-22) | 15 (9-27) ^*^^*^ | 14 (8-28) ^*^ |
|  | E_2_ (pM) | 123 (89-157) | 121 (82-150) | 122 (94-162) |
|  | TT (nM) | 11 (7-16) | 9 (6-14) ^**^ | 9 (7-15) |
| Male (N) |  | 296 | 76 | 68 |
|  | FSH (IU/L) | 18 (12-35) | 31 (14-43) ^*^ | 24 (15-42) ^*^ |
|  | LH (IU/L) | 10 (7-19) | 12 (7-25) | 13 (8-25) ^*^ |
|  | E_2_ (pM) | 129 (102-163) | 133 (102-154) | 130 (99-169) |
|  | TT (nM) | 12 (9-17) | 12 (8-14) | 10 (7-15) |
| Female (N) |  | 41 | 21 | 7 |
|  | FSH (IU/L) | 63 (51-76) | 69 (57-83) ^#^ | 106 (83-118) ^**^ |
|  | LH (IU/L) | 27 (21-35) | 27 (20-39) | 45 (34-51) ^*^ |
|  | E_2_ (pM) | 53 (43-79) | 54 (43-70) | 82 (27-101) |
|  | TT (nM) | 1.0 (0.7-1.4) | 1.0 (0.8-1.3) | 1.1 (0.6-1.6) |

Data were shown as median with interquartile range (25th percentile to 75th percentile). ^*^ p<0.05, ^**^ p<0.01 against normal cognition. ^#^ p<0.05 against vascular dementia. E_2_: estradiol; FSH: follicle-stimulating hormone; LH: luteinizing hormone; TT: total testosterone.
